# Carrot or Stick? Modelling How Landowner Behavioural Responses Can Cause Incentive-Based Forest Governance to Backfire

**DOI:** 10.1371/journal.pone.0077735

**Published:** 2013-10-30

**Authors:** Kirsten A. Henderson, Madhur Anand, Chris T. Bauch

**Affiliations:** 1 Department of Mathematics and Statistics, University of Guelph, Guelph, Ontario, Canada; 2 School of Environmental Sciences, University of Guelph, Guelph, Ontario, Canada; 3 Department of Applied Mathematics, University of Waterloo, Waterloo, Ontario, Canada; National Research & Technology Council, Argentina

## Abstract

Mitigating the negative impacts of declining worldwide forest cover remains a significant socio-ecological challenge, due to the dominant role of human decision-making. Here we use a Markov chain model of land-use dynamics to examine the impact of governance on forest cover in a region. Each land parcel can be either forested or barren (deforested), and landowners decide whether to deforest their parcel according to perceived value (utility). We focus on three governance strategies: yearly incentive for conservation, one-time penalty for deforestation and one-time incentive for reforestation. The incentive and penalty are incorporated into the expected utility of forested land, which decreases the net gain of deforestation. By analyzing the equilibrium and stability of the landscape dynamics, we observe four possible outcomes: a stationary-forested landscape, a stationary-deforested landscape, an unstable landscape fluctuating near the equilibrium, and a cyclic-forested landscape induced by synchronized deforestation. We find that the two incentive-based strategies often result in highly fluctuating forest cover over decadal time scales or longer, and in a few cases, reforestation incentives actually decrease the average forest cover. In contrast, a penalty for deforestation results in the stable persistence of forest cover (generally >30%). The idea that larger conservation incentives will always yield higher and more stable forest cover is not supported in our findings. The decision to deforest is influenced by more than a simple, “rational” cost-benefit analysis: social learning and myopic, stochastic decision-making also have important effects. We conclude that design of incentive programs may need to account for potential counter-productive long-term effects due to behavioural feedbacks.

## Introduction

Increasing human population, agricultural land-use and consumption of forest by-products threaten forest conservation and underscore the importance of effective governance [1]–[3], particularly when environmental services are considered, such as climate stabilization, water purification and ecological stability [4]. Introducing an economic incentive for individuals to maintain forested land with the intention of reducing the discrepancy between the value of ecological services and the market value of timber has been suggested as a mechanism to increase forest cover [5], [6]. For example, recently, the United Nations Framework Convention on Climate Change (UNFCCC) implemented REDD+ (Reducing Emissions from Deforestation and Forest Degradation), in which developing countries receive payments from corporations, nongovernmental organizations, and individuals for a demonstrated reduction in carbon emissions [7], [8]. In order to receive compensation, there must be noticeable improvements in forest protection, sustainable forest management, and/or enhancement of carbon stocks [9]. The developing countries' governments devise their own strategies to improve any or all of the above mentioned requirements [10]. REDD+ increases the global market value of forested land by placing a value on carbon stocks and uses incentives to protect critical ecosystems [10]. In the same vein, Costa Rica established payment for environmental services (PES) in 1997 and the program now successfully contributes to forest regeneration and reforestation efforts by providing private landowners with an economic incentive for ecosystem services enhancement [11].

Despite its successes, many criticisms of REDD+ persist. Concerns arise over insufficient local participation, lack of coherence between landowners and those making decisions, and applying a “one size fits all” solution under varying environmental and socio-economic conditions [12]. Also, REDD+ does nothing *per se* to change the quality of a government or its ability to enforce regulations [13], [14]. Indeed, Umemiya *et al*. reported that lower quality of governance at the national level is associated with higher deforestation rates [15]. Quality of government was defined by the involvement of citizens, stability of the government, quality of public services, ability to promote regulations, enforcement ability, and control of corruption and violence [15].

In the context of forest management, as well as more generally, it has been suggested that effective governance of natural resources requires the following elements: 1) collective-choice arrangements, which incorporates all affected individuals in decision-making on the appropriation and provision rules [16]–[18], 2) penalties that reflect the severity, frequency, and context of the violation [17], 3) a hierarchy of governance to promote policies and regulations, as well as mediate conflicts [2], [15], [17], [19], and 4) participating members who monitor the situation and control corruption [15], [19]. When one or more of these elements is absent (e.g. due to lack of enforcement mechanisms, insufficient information, or human error) myopic decision-making can lead to a cascade of uncooperative behaviour and exploitation of the commons, resulting in resource shortages [20]. For example, decentralizing forest governance responsibilities to localities without also transferring significant decision-making powers to them can stifle local interest in forest governance activities [21].

It has also been suggested that the relative effectiveness of implementing economic incentives versus relying on social norms as pathways to forest conservation depends on which cognitive processes are involved in decision-making [5]. Because forests regenerate slowly, decision-makers' accurate knowledge of present versus future gains is critical for appropriate forest use and conservation practices. Accordingly, short-term decision-making may result in forest destabilization and overuse of resources, due to landowner emphasis on reaping short-term benefits. Since economic gains from deforestation are the driving force behind a landowner's decision to deforest, providing economic incentives to landowners who conserve forest can – in principle – help bridge the gap between short-term and long-term decision-making [5].

Mathematical models have been used to capture the interplay between forest dynamics, landowner forestry practices, and determinants of landowner decisions [16], [22]–[24]. In these coupled human-environment systems models, landowners deciding whether to deforest or conserve are often assumed to make the decision that produces the largest expected utility under current conditions [16], [22], [23]. Utility reflects an individual preference, including both financial gain from selling timber, biofuels and non-timber forest products (utility of deforestation) as well as the less tangible benefits of ecological services (utility of conservation) provided by intact forests [22], [25]. Under the assumption of best-decision practices, landowners understand the trade-offs involved in maximizing present utility versus reaping future gains. However, under the assumption of myopic decision-making, landowners are more likely to seek instant rewards without considering the long-term profit, or long-term damages caused from loss of ecosystem services or the consequences to surrounding landowners [23]. A model by Satake *et al*. shows that including realistic features of populations such as social learning, myopic decision-making, and uncertainty regarding true utilities can elicit a wide range of outcomes, including both stable and unstable states, and forested versus deforested conditions [23]. The same model also allows individuals to use knowledge gained from experiences, which is predicted to lead to better forest management and controlled deforestation [23].

There is still ample debate about the relative roles of conservation versus reforestation, and how to design incentives for each to maintain long-term forest cover in targeted regions. Despite this, the impact of governance is rarely considered in coupled human-environment system models of forest dynamics and harvesting. Here, we build on a model by Satake *et al*. [23] to analyze the influence of governance – in the form of either economic incentives or penalties – on forest management. We then compare the results of our model with the definition of a quality government stated by Umemiya *et al*. [15] to measure the effectiveness of the governance strategies simulated. Our model can be interpreted in terms of both centralized and decentralized governments, where the rewards or penalties are determined on a national level or local level. We consider three governance strategies based on currently employed strategies in different regions of the world: incentive for (1) forest conservation or (2) reforestation, either in the form of a direct payment [11] or a tax benefit [21], [26], or (3) penalty for deforestation, usually in the form of a tax [27] or a fine for breaking contract terms [28]. Our objective is to explore whether governance applied to a population where social learning plays a large role in decisions leads to improved decision-making and more effective forest management. We hypothesize that greater governance intervention (*i.e*. greater incentive or heavier penalty) should yield higher and more stable forest cover.

## Model Description

We expand on the model by Satake *et al*. by including governance [23]. Since utility is a primary factor in a landowner's decision to deforest, we incorporate governance into the individuals' expected utility, where the amount and effectiveness of government is controlled by a model parameter. We implement the following Markov chain model as an agent-based simulation, using Netlogo 5.0.2 [29]. All model parameters and variables appear in Table 1.

**Table 1:** Description and range for parameters/variables of the model.

	Symbol	Description	Range	Source
**Parameters**	*β*	Positive constant for degree of stochasticity in decision-making. Large values of *β* indicate decisions based on certainty of reward. This parameter is absorbed into *b'*, *c'* and *g'* through rescaling.	0-∞	[23]
	***α***	Forgetting coefficient. *α* represents the extent to which past knowledge is used in current decision-making.	0–1	[23]
	***μ***	Transition probability from forested to deforested state. 1/*μ* represents the expected number of years needed for forest recovery.	0.01–0.5	[23]
	***b*** *'*	Positive constant for utility received from ecosystem services of land of land in the forested state. *b'* = *bβ*	0–5 000	modified from [23]
	***c*** *'*	Profit from deforestation. *c'* = *cβ*	0–20 000	modified from [23]
	***g*** *'*	Positive constant for government intervention represented by an incentive or a penalty.	0–20 000	Eqs. (9,11,13)
**Variables**	*S_i_*(*t*)	State variable of the *i*th land parcel in year *t*. Forested states represented by 1, deforested state represented by 0.	0,1	[23]
	*u_i_*(*t*)	Actual utility received by landowner *i* in year *t*.	0–20 000	modified from [23]
	*r*(*t*)	Rate of deforestation in year *t*.	0–1	modified from [23]
	*π_F_*(*t*)	Experienced utility of forest conservation in year *t*, averaged in society.	0–5000	modified from [23]
	*π_D_*(*t*)	Experienced utility of deforestation in year *t*, averaged in society.	0–10 000	modified from [23]
	***x(t)***	Density of forested land in year *t*.	0–1	modified from [23]
	***y(t)***	Density of deforested land in year t.	0–1	modified from [23]
	***w(t)***	Density of land that has just been reforested in year *t*.	0–1	Eq. (17c)
	***V_D_(t)***	Expected utility of deforestation in year *t*.	0–10 000	modified from [23]
	***V_F_(t)***	Expected utility of forest conservation in year *t*.	0–15 000	modified from [23]
	*π_gF_*(t)	Experienced governance incentive in year *t*, averaged in society.	0–10 000	Eq. (8)
	*u_g_*(t)	Governance utility received by landowner *i* in year *t*.	0–20 000	Eqs. (9,11,13)

After rescaling and substitutions, only 5 variables and 5 parameters remain in the model – these symbols are indicated using bold typeface in the table.

### 2.1 Two-state Markov chain: deforestation and forest recovery

**Figure 1:**
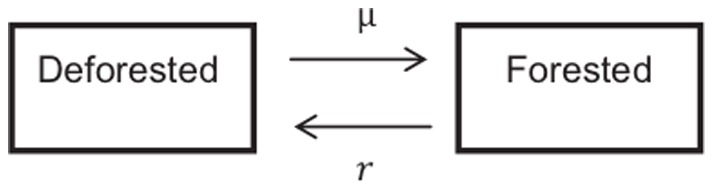
Transition between forested and deforested states. The transition of a land parcel from the forested state to deforested and vice versa. *r*(*t*) is the deforestation rate, controlled by the net gain of deforestation. *μ* is the forest recovery rate.

The basic model assumes that a forest is composed of *N* land parcels where each landowner *i* is responsible for parcel *i* [23]. The parcel can either be forested or deforested (Figure 1). *S_i_*(*t*) is the state of the *i*th parcel in year *t*:
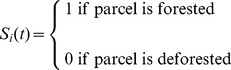
(1)


The forest recovery rate, *μ*, is the probability per year that the parcel transitions from a deforested state to a forested state (hence, the average time required for a forest to recover is 1/*μ*). The utility received by a landowner depends on the state of their land; *u_i_*(*t*) is the actual utility received by landowner *i* in year *t*:
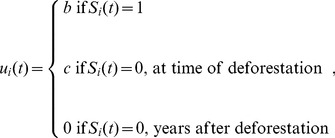
(2)where *b*>0 is the utility associated with ecosystem services for a parcel in the forested state, and *c*>0 is the utility associated with deforesting (the financial gain from the resulting timber). The utility after deforestation is 0 since there is no financial gain from bare land (we assume bare land is not economically viable for farming) and for simplicity, ecosystem services of deforested land do not contribute to utility [23].

### 2.2 Individual decision about deforestation

A landowner decides whether to deforest based on the net gain of deforestation
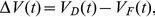
(3)where *V_D_*(*t*) is the expected utility of deforestation and *V_F_*(t) is the expected utility of conserving the parcel as forest in year *t* (derived from Eq. (5)). A forested parcel is deforested in year *t* with probability
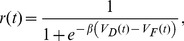
(4)where r(t) is the transition probability from forested to deforested state in year t, and β is a positive constant such that β→∞ represents purely deterministic decision-making (where landowners always deforest when VD(t)-VF(t)>0) and smaller values of β imply stochastic decision-making (where landowners deforest with probability less than 100% even if VD(t)-VF(t)>0). To simplify the analysis we reduce the number of parameters by absorbing β into Eq. (2) through the rescaling b'  =  bβ and c'  =  cβ. The parameters b' and c' now range from (0,∞). As a result, the probability of deforesting becomes 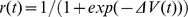. After rescaling, the relative magnitude of b' versus c' as reflected in ΔV(t) simultaneously represents the degree of stochasticity in decision-making, as well as the difference between utilities received for deforesting versus maintaining a forested parcel. As ΔV(t) approaches infinity (larger b' and c'), the landowner's decision is completely determined by the difference in utilities, either because decision-making is sufficiently deterministic or because the inherent difference in utilities is sufficiently large. However, for smaller values of ΔV(t) (smaller b' and c') landowners are more likely to disregard the net gain from deforestation and make decisions based on alternative assumptions, i.e. stochastic decision-making.

In the absence of governance, *V_D_*(*t*) and *V_F_*(*t*) take the form
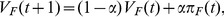
(5a)

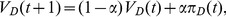
(5b)where 0<*α*<1 is the “forgetting coefficient” representing the extent to which past knowledge is used in current decision-making (*α* = 0 incorporates only prior knowledge whereas *α* = 1 is a decision based solely on current information), and where *π_F_*(*t*) and *π_D_*(*t*) represent knowledge of utilities gained from other landowners' experiences at time *t* regarding the utility for forested and deforested states respectively. These experienced utilities are a function of land states and actual utilities according to



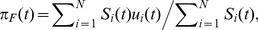
(6a)

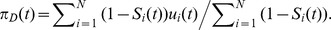
 (6b)

Hence these equations describe a population where landowners learn expected utilities from the experiences of other landowners, who share their information.

### 2.3 Forms of governance

#### 2.3.1 Governance as a yearly incentive for conservation

Under yearly incentives for maintaining a parcel as forested, the individual's expected utility for a forested state is augmented by a governance incentive:
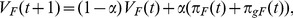
(7)where *π_gF_*(*t*) represents the experienced governance incentive at time *t*:



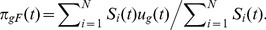
(8)Hence we assume that the knowledge gained includes knowledge of the governance incentive issued to other landowners maintaining forested parcels.

Including a governance parameter increases the utility received by landowners maintaining parcels in the forested state. *u_g_*(*t*) is the governance utility received by the each landowner *i* in each year *t*:
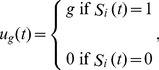
(9)where *g* ∈ [0,1] is the incentive given to landowners for maintaining a forested parcel; *g = *0 represents governance without restrictions on deforestation (*i.e*. no government structure present) and *g = *1 represents the greatest involvement (*i.e*. largest fine). For consistency with the rescaling of *b* and *c,* we also rescale *g* according to *g'*  =  *gβ*. The rescaled parameter *g'* ranges from (0,∞). The landowner receives an incentive each year that the parcel is forested. A landowner choosing to deforest receives no governance utility.

Here, *g'* is parameterized as a percentage of the utility received from deforestation. In real populations, it has been found that incentives that contribute 1–2% of a household's income are ineffective at promoting forest conservation [30]. The average landowner receives less than 10% of their income through incentives from Costa Rica's payments for environmental services [31]. In our analysis we will consider values of *g'* ranging from 0 to 50% of gains from deforestation.

The individuals' expected utility *V_D_*(*t*) for a deforested parcel is unchanged (Eq. (5b)).

#### 2.3.2 Governance as a one-time penalty for deforestation

In this case, the individuals' expected utility for maintaining a forested parcel is unchanged (Eq. (5a)).

However, governance decreases the expected utility received by landowners at the time of deforestation through a penalty:
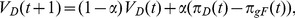
(10)where *π_gF_*(*t*) is given by Eq. (8) but now *u_g_*(*t*) is the governance penalty received by the landowner *i* in year *t*:
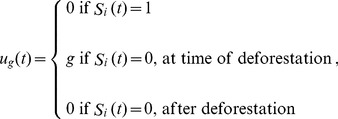
(11)and where g is the penalty incurred for deforesting at time t. Again, we rescale g according to g'  =  gβ. We explore penalties between 0–50% of the landowners' gains from deforestation.

#### 2.3.3 Governance as a one-time incentive for reforestation

Governance applied as a one-time incentive to reforest increases the utility received by landowners only at the time of reforestation:
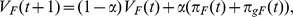
where *π_gF_*(*t*) is given by Eq. (8), but now *u_g_*(*t*) is the reforestation incentive received by landowner *i* in year *t*:
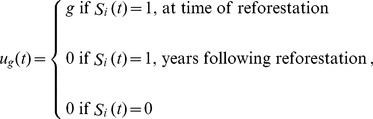
(12)where g ∈ [0,2] is the incentive given to landowners to reforest. Note the maximum possible incentive to reforest (g = 2) is greater than the maximum possible incentive to conserve a forested parcel (g = 1), since the incentive to conserve must be given yearly whereas the incentive to reforest need be given only once, when the forest recovers. Here we rescale g as we do for the yearly incentive for conservation in Eq. (9), where g' = gβ. The parameter g' ranges from 0 to ∞.

The individuals' expected utility *V_D_*(*t*) for a deforested parcel is unchanged (Eq. (5b)).

### 2.4 Landscape dynamics

We define *x*(*t*) as the density of forested parcel and *y*(*t*) as the density of just-deforested parcels (parcels that were forested in the previous time step). Hence, the density of parcels that were deforested more than a year ago and have not recovered is 1-*x*(*t*)-*y*(*t*). The landscape dynamics are given by
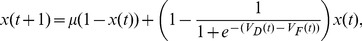
(13a)

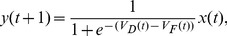
(13b)where *μ* is the probability per yearly time step that a deforested parcel becomes forested (*i.e.* the forest recovery rate).

### 2.4.1 Governance as a yearly incentive for conservation

Under yearly incentives, the landscape dynamics can be expressed using Eqs. (13a, 13b) as:
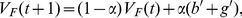
(14a)

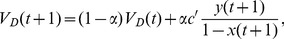
(14b)


#### 2.4.2 Governance as a one-time penalty for deforestation

Under a one-time penalty for deforestation, the landscape dynamics can be expressed using Eqs. (13a, 13b) as:
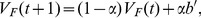
(15a)

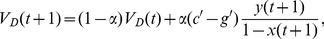
(15b)


#### 2.4.3 Governance as a one-time incentive for reforestation

For this scenario, we introduce *w*(*t*), the density of land that has just been reforested. The landscape dynamics are given by:
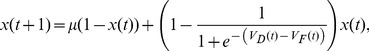
(16a)

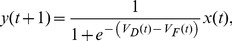
(16b)




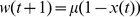
(16c)


Using Eqs. (16a, 16b, 16c), the expected utilities become
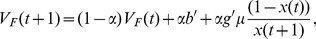
(17a)

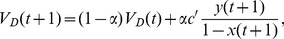
(17b)


## Results

### 3.1 Governance as a yearly incentive for conservation

There exists a single positive equilibrium for the landscape dynamics Eqs. (13a, 13b, 14a, 14b).


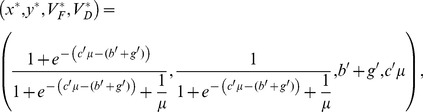
(18)

The expression for *x* ^*^ from Eq. (18) yields the ratio of forested to deforested land at equilibrium:


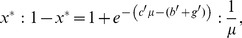
(19)

In this case the results are essentially as in Satake *et al.* [23], except *b'* has now been replaced by *b'*+*g'.* The net utility gain from deforestation at equilibrium is given by
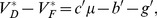
(20)


If *g'>c'* µ*-b'*, the net utility gain of deforestation is negative (the loss of ecosystem services *b'* and the government incentive *g'* exceeds the gain from timber extraction *c'*), hence we expect to see a greater density of forested parcels *x* ^*^ as long as utility is a factor in decision-making (*i.e*. *b'*, *c'* and *g'* are sufficiently large) (Eq. (19)). Indeed, for large values of utility received and *g'>c'*µ*-b'*, the landscape is a stationary-forested equilibrium; if *g'<c'*µ*-b'* the landscape is stationary deforested (Eqs. (18,19)).

**Figure 2:**
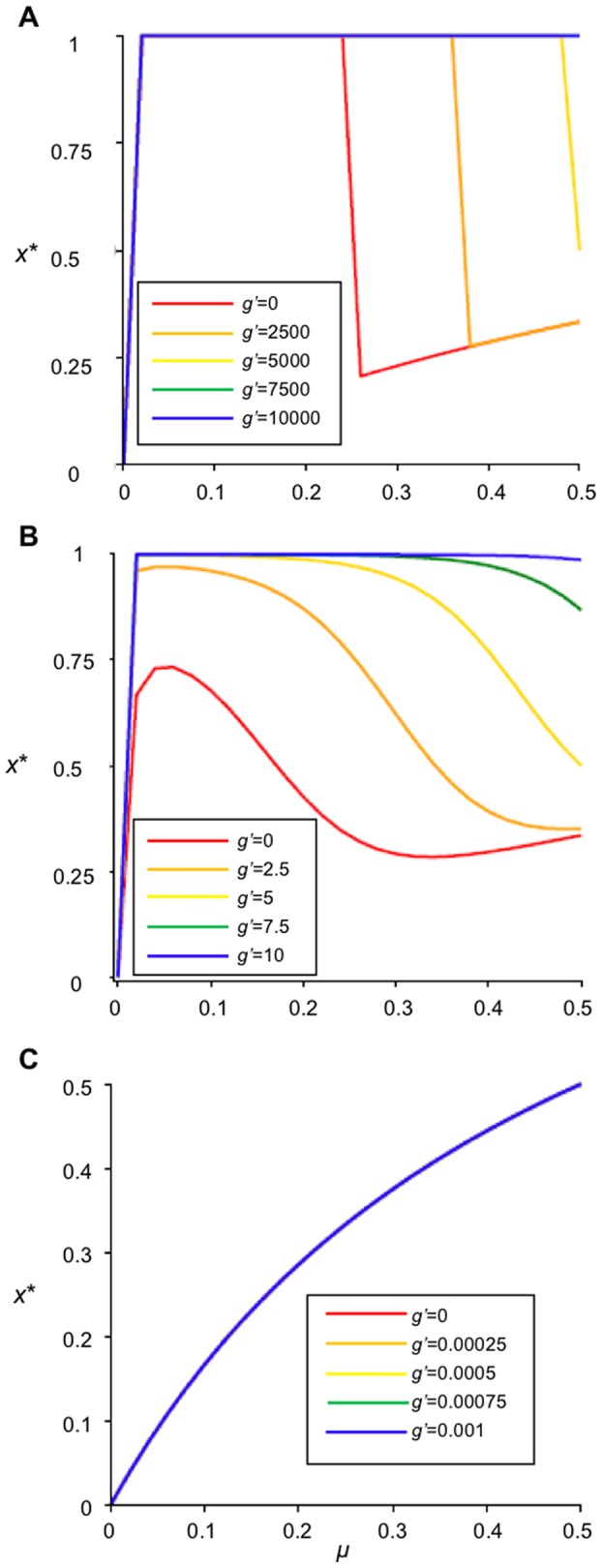
Effects of governance *g'* as a yearly incentive for conservation and forest recovery rate *μ*. Plot representing the fraction of forested land at equilibrium (x*) versus forest recovery rate (*μ*). *g'* is the governance coefficient, representing an incentive given on a yearly basis to landowners choosing forest conservation. (A) illustrates landowners employing best choice decision-making with larger values of utility received (*b'* = 5 000, *c'* = 20 000). (B) represents *b'* = 5 and *c'* = 20 (C) illustrates landowners employing probabilistic decision-making with smaller values of received utility (*b'* = 0.0005 and *c'* = 0.002). As *g→* 10 000 (*i.e.* greatest incentive to maintain forest cover) forest conservation prevails, independent of *μ*.

For deterministic decision-making (*b', c', g'*→∞, where the landowners' decision is based solely on the net utility gain from deforestation), this observation is supported by plots of equilibrium forest cover x* versus *μ* with various values of *g'* (Figure 2A). As *g'* increases, x* also increases, regardless of the value of the forest recovery rate *μ.* Figure 2A, the landscape is heavily forested at all values of *μ*; this corresponds to a stationary-forested landscape for the equilibrium value described in Eq. (18). When *b'* and *c'*→∞, the time in the forested state is also infinitely long (x* = 1) if *g'>c'*µ*-b'*.

The net gain of deforestation (Eq. (20)) is an increasing function of the forest recovery rate *μ*: when *μ* is higher, deforested parcels become reforested more quickly, meaning that the landowner can harvest the timber more frequently and hence gain more utility from deforesting. When *b', c', g'*→∞ and for certain ranges of *μ*, this effect can be strong enough to cause the equilibrium forest cover x* to actually decrease (instead of increase) with increasing forest recovery rate *μ* (Figure 2A).

For intermediate values of utility received and government incentive (Figure 2B), predictions are qualitatively unchanged from the scenario: increasing *g'* always increases x*, and increasing *μ* will sometimes cause a decrease in x*.

However, as *b', c*' and *g'* approach 0, landowners increasingly disregard the net utility gain of deforestation and instead adopt a purely probabilistic (stochastic) approach to decision-making; this results in an increasing relationship between x* and *μ*. For smaller values of utility received and government incentives, the time spent in the forested state is greatly reduced (x*<0.5 for all *μ*; Figure 2C).

Eq. (4) and Eq. (20) can be used to find the best choice whether to deforest under social learning and yearly incentive governance:
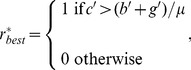
(21)where *c'* represents the short-term returns from deforestation and (*b'+g'*)/*μ* represents the loss of forested land utility including the yearly governance incentive; 1 is indicative of the best choice about deforestation.

Analyzing the dependence of x* on model parameters does not tell us anything about how the stability of x* depends on model parameters. To explore model stability we simulate the model and generate time series for *x*(*t*) for various values of *μ*, *g'*, and *α* (the forgetting coefficient). For these simulations, we will explore how dynamics change between low *α* (long-term memory) and high *α* (short-term memory), in addition to small *μ* and large *μ*.

**Figure 3:**
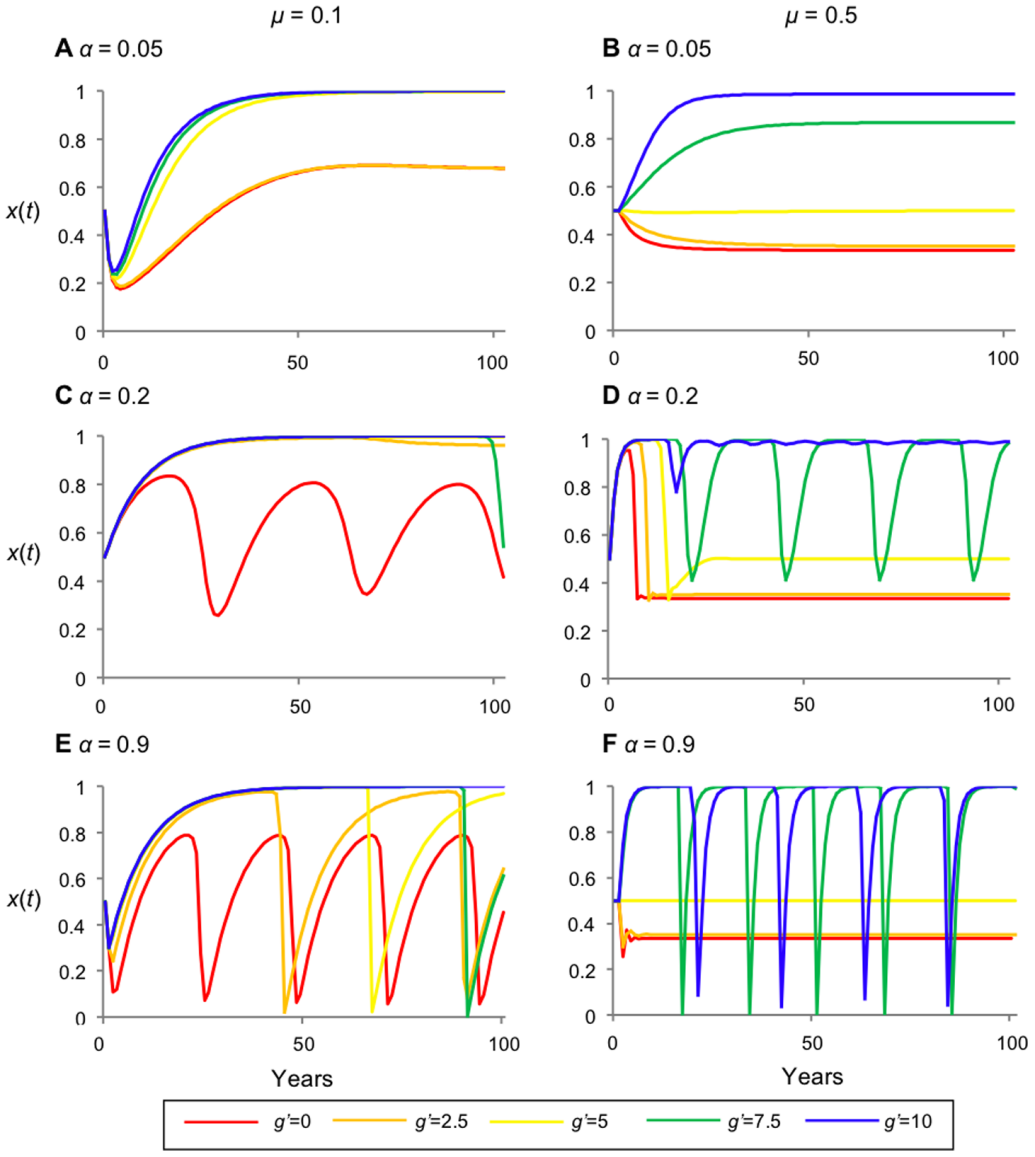
Destabilizing effects of forest recovery rate *μ* and short-term memory *α* under governance as a yearly incentive *g* for conservation. Time series representing the fraction of forested land (x(t)) under yearly incentive for conservation. (A) has a forgetting coeffiecient of *α* = 0.05 and recovery rate *μ* =  0.1; (B) *α* = 0.2 and *μ* =  0.1; (C) *α* = 0.9 and *μ* =  0.1; (D) *α* = 0.05 and *μ* = 0.5; (E) *α* = 0.2 and *μ* = 0.5; (F) *α* = 0.9 and *μ* =  0.5. Other parameters are *b'* = 5, *c'* = 20. *α* increases the amplitude of oscillations and *μ* increases the frequency; as *g'* increases so does the forest cover, but not necessarily stability.

The equilibrium x* is independent of *α*, however, *α* has a significant impact on the stability of x* (Figure 3). When *α* and *μ* are small (*i.e. α* = 0.05 and *μ* = 0.1) the parcels converge, for all values of *g'*, to a stable forested landscape (*i.e*. stationary-forested landscape) (Figure 3A); the landscape is represented almost entirely of parcels in the forested state. When *μ* is larger (*α* = 0.05 and *μ* = 0.5), dynamics converge either to stationary forested or stationary de-forested landscapes (Figure 3B). However, as *α* increases and decisions are based on shorter-term memory, the equilibrium destabilizes, creating large-amplitude oscillations – synchronized deforestation – in forest cover (Figure 3E, H). Since forest density *x*(*t*) depends on the net gain of deforestation, which incorporates the expected utilities, the same fluctuation cycles are observed in the expected utilities (results not shown).

Increasing *μ* increases the frequency of oscillations. This is to be expected since larger values of *μ* mean forest cover regenerates more quickly, subsequently allowing landowners to deforest a greater density of parcels at a faster rate. However, small values of *g'* (*i.e. g'*<5, weaker governance incentive) counteract the destabilizing effects of *μ* on forest density, allowing the system to converges to a stationary-deforested landscape. Oscillations in forest cover are largest and most frequent when both α and *μ* are large.

The effect of increasing the incentive *g'* can be either to stabilize – or more interestingly to destabilize – dynamics (Figure 3). Increasing *g'* sufficiently appears to stabilize dynamics (decrease the amplitude of oscillations) when *α*<0.2 because the incentive is sufficient to motivate more landowners to arrest deforestation, leading to *x*(*t*) = 1 (Figure 3C). However in other cases, increasing *g'*, in combination with large *α* and *μ* values, causes instability (Figure 3D, F). Indeed, local stability analysis Matrix (S1) in Supporting Information S1 shows that the equilibrium is unstable for *α* = 0.2, *μ* = 0.5 and *g'*>5. This occurs because without the incentive, the system convergences to a stationary deforested state, but when governance is added, landowners begin to reconsider and some of them will start to reforest. However, this ends up leading to a classic “boom-bust” cycle under myopic decision-making: when *g'* is sufficiently large, forest cover surpasses the equilibrium due to the large incentive for maintaining forested parcels; once the forested land expands over nearly the entire landscape, the expected utility of deforested land surpasses the equilibrium utility (because, after enough time has passed, due to myopic decision-making, landowners consider only the recent experiences of landowners who deforested and experienced a quick gain from deforestation (*c'*>*b'*), but they fail to consider the yearly incentive for forest conservation and the long-term ecosystem services benefits, which actually produce a greater net gain in the long-term). This, in turn, encourages numerous landowners to simultaneously deforest. Not only does increasing *g'* result in destabilization by causing forest cover to overshoot x*, but also in the trough of the “boom-bust” cycle, forest cover can be lower than it would have been without any governance at all (compare *g'* = 0 to *g'* = 7.5, 10 in Figure 3F). Such low levels of forest cover would introduce effects like soil erosion and extreme habitat fragmentation, leading to a strongly counter-productive effect of governance. This dynamic depends upon myopic decision-making; in contrast, if landowners employ past knowledge (*i.e*. *α*→0), they continue to conserve their forested parcels, remembering that there is a period of decreased return immediately after deforestation [23].

If the expected utility of forested land is independent of forest cover (*i.e*. *α* = 0), the system is always stable.

### 3.2 Governance as a one-time penalty for deforestation

In this case there exists a single positive equilibrium for the landscape dynamics Eqs. (13a, 13b, 14a, 14b).


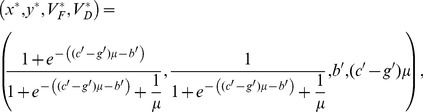
(22)

In this case the results are essentially as in Satake *et al.* [23], except *c'* has now been replaced by *c'-g'.* The net utility gain from deforestation at equilibrium is given by
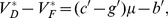
(23)


When (*c'*-*g'*)*μ*<*b'* (or *g'>c'-b'/μ*), the net gain of deforestation is negative, which increases the fraction of forested parcels.

Comparing Eqs. (23) and (20) reveals an important asymmetry between the scenarios of yearly incentives to retain a forested patch versus one-time penalties for deforesting. In the former case, *g'* is simply added to the value of *b'* and is applied each year, but in the latter case, *g'* is subtracted from *c'* and is applied only in the year that the decision to deforest is made; this will have implications for stability under myopic decision-making.

Equations (4) and (23) are used to find the best choice for deforestation under social learning and deforestation penalty governance:


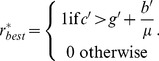
(24)

These results are similar to Eq. (21), except *g'+b'/μ* represents the penalty incurred for deforestation and the loss of forested utility; 1 represents the circumstances under which landowners reap the greatest rewards. *b', c'* and *g'* influence the landowners' choice about deforestation, similarly to incentive for forest conservation governance except the rate of deforestation is more sensitive to changes in (*c'-g'*) *μ*-*b'* and the time spent in the forested state is greater for *g'* >*c'-b'*/µ.

**Figure 4:**
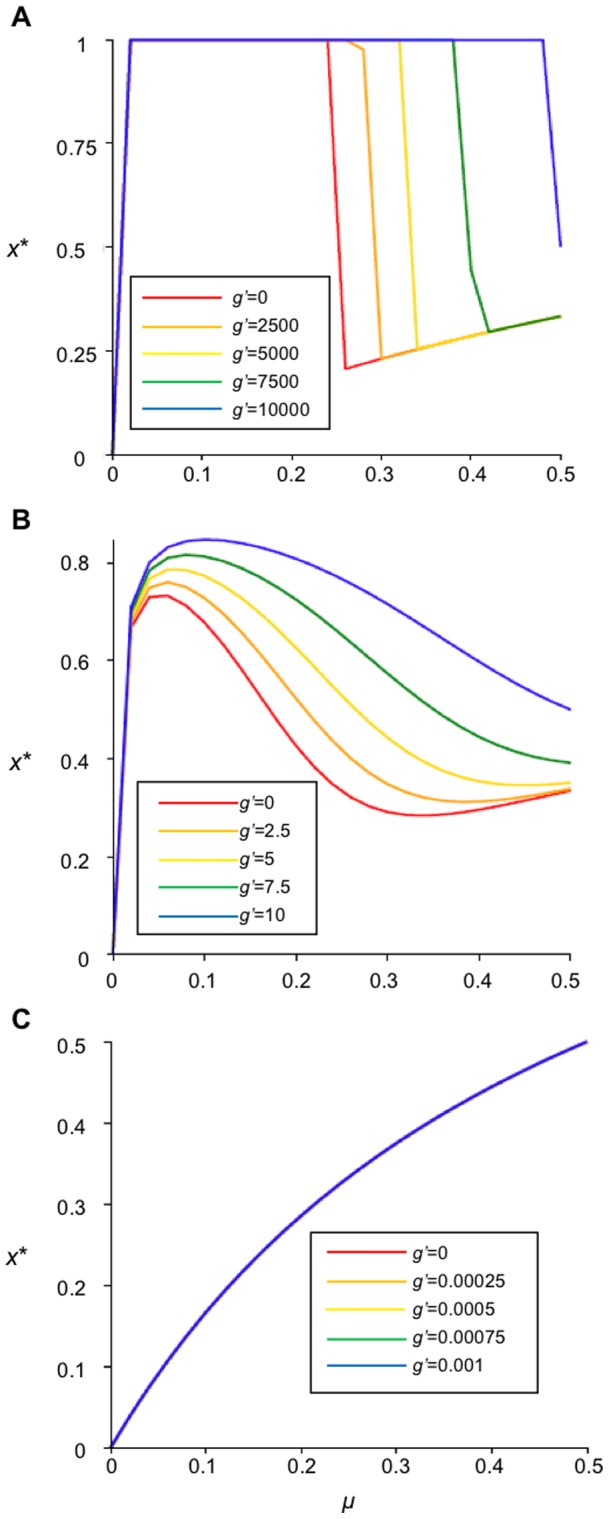
Effects governance *g'* as a one-time penalty for deforestation, and forest recovery rate *μ*. Plot representing the fraction of forested land at equilibrium (x*) versus forest recovery rate (*μ*). g is the governance coefficient, representing a penalty at the time of deforestation. (A) illustrates landowners employing best choice decision-making with larger values of utility received (*b'* = 5 000, *c'* = 20 000). (B) represents *b'* = 5 and *c'* = 20 (C) illustrates landowners employing probabilistic decision-making with smaller values of received utility (*b'* = 0.0005 and *c'* = 0.002). As *g'→*10 000 (*i.e.* greatest incentive to maintain forest cover) forest conservation prevails, independent of *μ*.

The relationship between x*, *g'*, and *μ* is similar to that for the case of yearly incentives to maintain forest (Figure 4 versus Figure 2): increasing *g'* always increases x*, and there are ranges of *μ* values for which x* will decline as *μ* increases. The main difference is that governance is generally somewhat less successful at increasing x* (compare Figure 4A to Figure 2A, and especially Figure 4B to Figure 2B). This occurs because *g'* has a similar magnitude in the two cases, but *g'* is applied every year in Figure 2 but only in the year of deforestation in Figure 4, hence the total amount of intervention over time is less.

**Figure 5:**
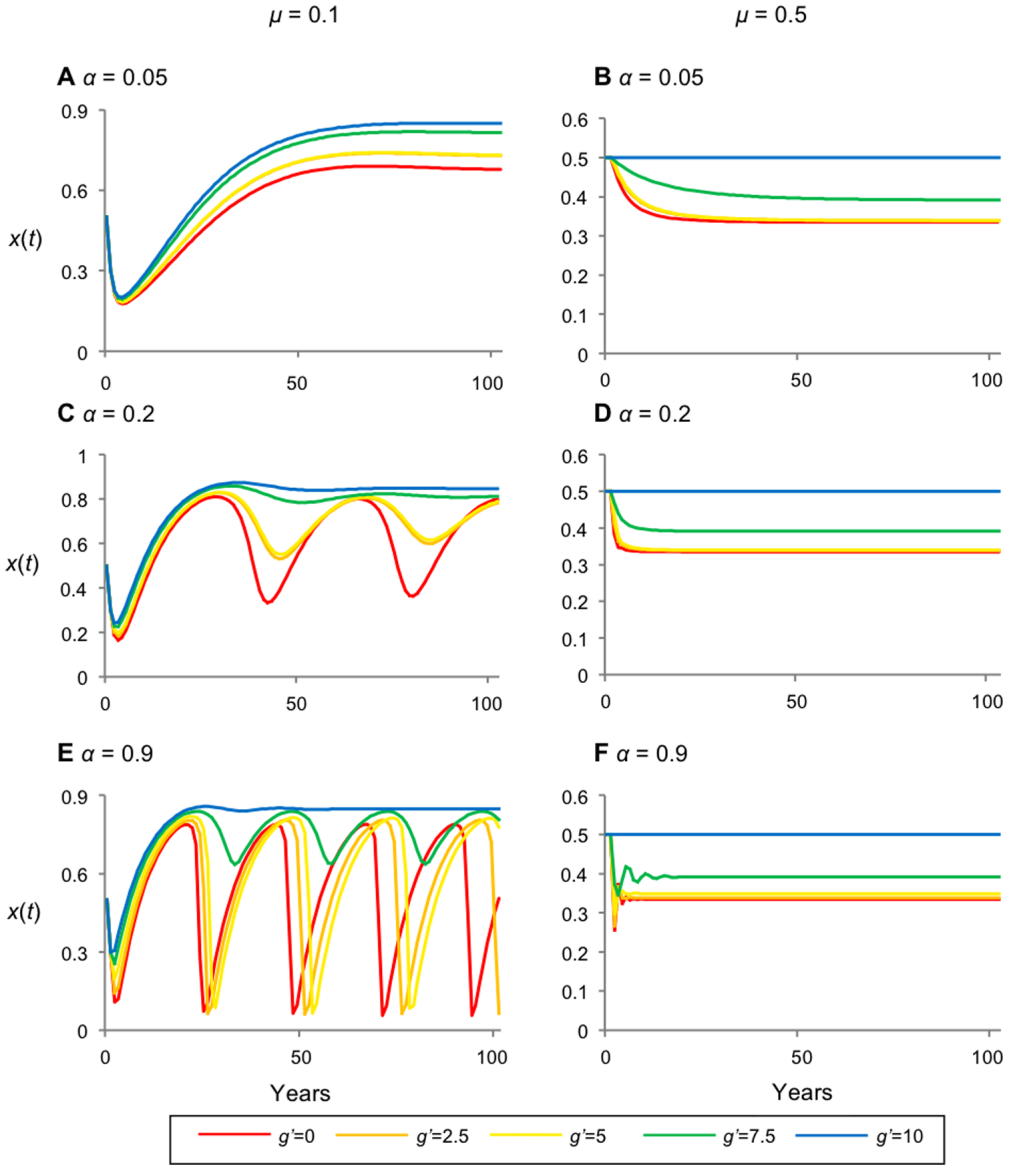
Stabilizing effects of forest recovery rate *μ* and large penalties *g'* under governance as a one-time penalty for deforestation. Time series representing the fraction of forested land (x(t)) under penalty for deforestation. (A) has a forgetting coeffiecient of *α* = 0.05 and recovery rate *μ* =  0.1; (B) *α* = 0.2 and *μ* =  0.1; (C) *α* = 0.9 and *μ* =  0.1; (D) *α* = 0.05 and *μ* = 0.5; (E) *α* = 0.2 and *μ* = 0.5; (F) *α* = 0.9 and *μ* =  0.5. Other parameters are *b'* = 5, *c'* = 20; increase in α, increases the amplitude of oscillations; increase in *μ* decreases the forest cover; increasing values of *g'* increase the forest cover and stability.

Comparing Figures 3 and 5 reveals both similarities and differences between the case of governance as a yearly incentive to conserve and governance as a one-time penalty to deforest. In Figure 5, as in Figure 3, we observe that dynamics tend to be less stable for myopic decision-making (*α* is higher). However, in Figure 5, increasing *g'* is always beneficial and has no counter-productive effects: it always boosts the average forest cover while also stabilizing dynamics even when *α* is high (by comparison, in Figure 3, increasing *g'* sometimes destabilizes dynamics). This difference occurs because the penalty occurs only at the time of deforestation, not in the distant past, hence myopic decision-making (large *α*) does not prevent landowners from being fully aware of the penalty incurred by deforestation, which makes landowners more hesitant to deforest. The stabilizing effects of the penalization are particularly obvious when the forest recovery rate is higher (*μ* = 0.5, compare Figure 5B, D, F to Figure 3B, D, F). However, the drawback of the one-time penalization approach is that the average forest cover can sometimes be lower (compare Figure 5B, D to Figure 3B, D) on account of *g'* only being applied once instead of continuously. The system is more likely to converge to the stationary-deforested landscape as *μ* increases, which emphasizes another difference between the two cases; in contrast, increasing the forest recovery rate *μ* tends to decrease stability under governance as a yearly incentive. Stationary-deforested refers to landscapes converging to states consisting of less than 50% forest cover. The types of instability are described in detail in Supporting Information S1.

In summary, governance as a one-time deforestation penalty generates less forest cover on average compared to governance as a yearly incentive, but forest cover is more stable over time, with no dangerous, intermittent crashes in forest cover. Also, increasing the forest recovery rate *μ* increases stability (completely nullifying the destabilizing effects of *α*) instead of decreasing it.

### 3.3 Governance as a one-time incentive for reforestation

**Figure 6:**
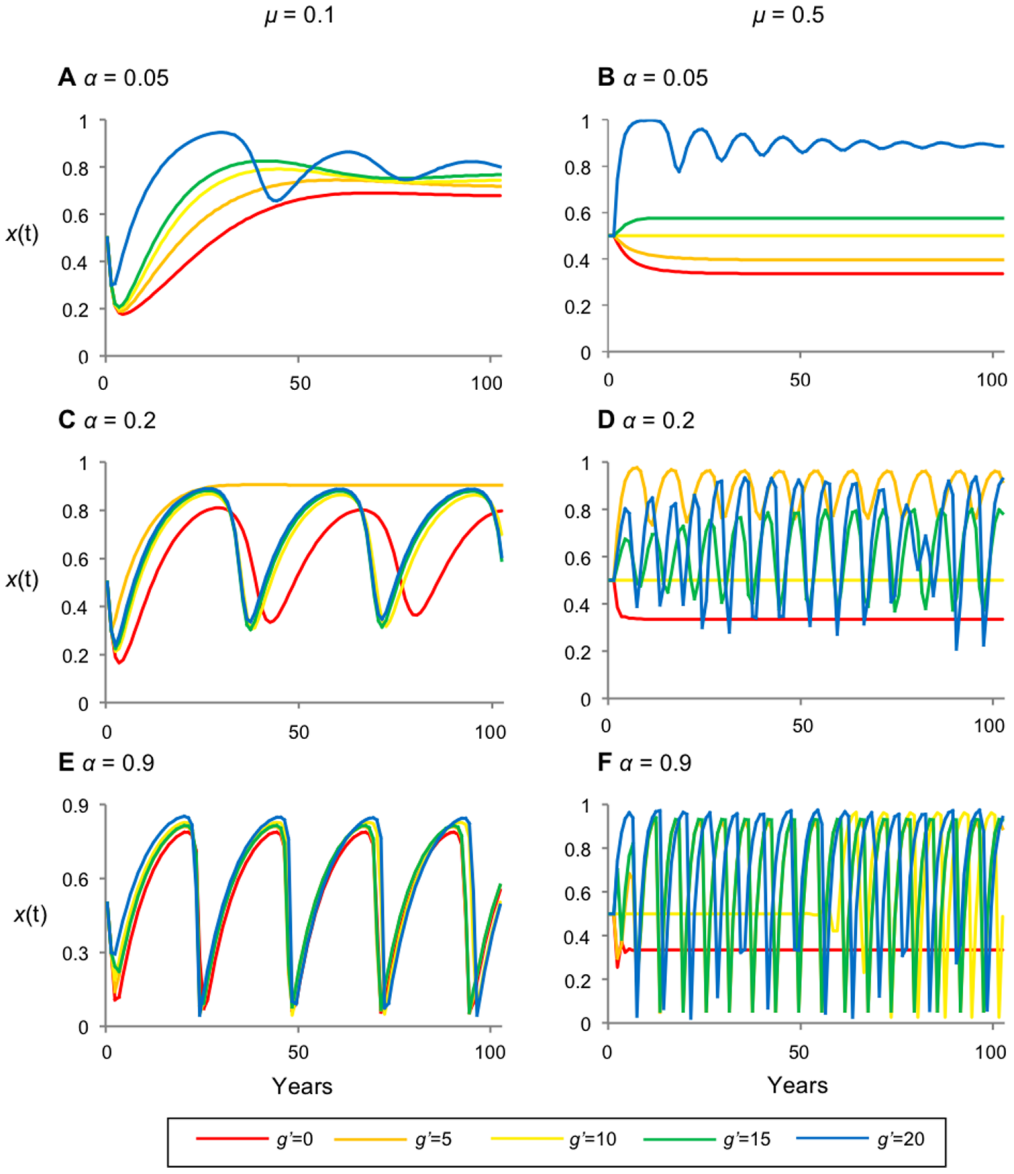
Destabilizing effects of forest recovery rate *μ* and short-term memory *α* under governance as a one-time incentive *g'* for reforestation . Time series representing the fraction of forested land (x(t)) under incentive for reforestation. (A) has a forgetting coeffiecient of *α* = 0.05 and recovery rate *μ* =  0.1; (B) *α* = 0.2 and *μ* =  0.1; (C) *α* = 0.9 and *μ* =  0.1; (D) *α* = 0.05 and *μ* = 0.5; (E) *α* = 0.2 and *μ* = 0.5; (F) *α* = 0.9 and *μ* =  0.5 *α* increases the amplitude of oscillations and *μ* increases the frequency; increasing values of g decrease stability. Other parameters are *b'* = 5, *c'* = 20.

In this scenario we were not able to identify a positive equilibrium for Eqs. (16a, 16b, 16c, 17a, 17b) when *g'*>0. However, numerical simulations suggest that forest cover should be less stable under governance as a one-time incentive for reforestation than under other forms of governance (Figure 6). Oscillations occur for a wider range of values for *g'*, *α*, and *μ* than for the other two forms of governance.

The impacts of changing *α* and *μ* on stability are similar under this form of governance: the amplitude of oscillations increases with increasing values of *α*; both the frequency and amplitude of oscillations increases with increasing *μ*. For a given value of *α* and *μ*, increasing the value of *g'* increases the magnitude of oscillations, resulting in synchronized deforestation. Since we are unable to determine the stability of the equilibrium under a one-time incentive for reforestation, we compare the results from the other two time series and their stability analysis to infer that too large an incentive induces synchronized deforestation and too little incentive generates the usual deforestation cycle created by a landowner's decision to deforest when forest cover is abundant. The value of *g'* must be calibrated to prevent either undesirable extreme.

Interestingly, for the case *α*  = 0.2 and *μ* = 0.1 (Figure 6C), increasing *g'* initially increases the average forest cover, until stable 100% coverage is reached when *g'* = 5, but as *g'* continues increasing, forest cover destabilizes again and the average forest cover decreases. Hence, beyond a certain point, the reforestation incentive actually decreases average forest cover and causes instability. In other cases (Figure 6E, F), incentives appear to have little effect on average forest cover, and also increase the propensity to oscillate.

This form of governance tends to destabilize dynamics because landowners initially gain from the reforestation incentive, then from the gains of timber extraction if they subsequently choose to deforest. This creates a cycle of high values for expected utilities of deforestation followed by high values for expected utilities of reforestation. As before, as the fraction of forested land approaches 1 the expected utility of deforested land increases, such that the yield from deforested land is greater than forested land; the value of timber products exceeds the value of ecosystem services motivating landowners to cut trees. However, in this case, once a parcel has been deforested, the one-time incentive to reforest encourages landowners to forest their land parcels as soon as possible due to an increase in the expected utility of forested land. The value of forested land only depreciates following the time step where the reforestation incentive is gained, while deforested land gains value as the forest cover increases. Hence, governance as an incentive for reforestation motivates landowners to deforest (gaining *c'*) and subsequently reforest (gaining *g'*), enabling landowners to deforest again, creating a continuous cycle motivated by short-term gains. Higher values of the forest recovery rate *μ* reinforce this motivation, making the system more prone to oscillation, or making existing oscillations more extreme (Figure 6).

## Discussion

When there is little incentive to ensure provision of ecosystem services, landowners in many circumstances choose to deforest [5], [24], [32]. However, properly designed governance structures can promote forest cover [33], [34]. Costa Rica was once a world leader in deforestation: from 1986 to 1991 the country saw forested land decline by 4.2% per year [11]. Through the PES program, forest regeneration has increased, typically via plantations, and Costa Rica has seen significant declines in deforestation rates [35]. In the northern region of Costa Rica the deforestation rate of natural forest declined from 1.43% per year before PES to 0.10% per year after the program went into effect [35]. Here we explored three forms of governance in a population of landowners making decisions according to expected utilities and social learning, and subject to the limitations of myopic decision-making. Our initial hypothesis that larger incentives and penalties always yield larger and more stable forest cover is not supported in our findings. In many cases, especially when decision-making was myopic, incentives destabilized dynamics, causing long-term fluctuations between states of densely forested land and deforested land. In a few cases, incentives caused a decrease in average forest cover. The decision to deforest is influenced by more than a simple, “rational” cost-benefit analysis: social learning, myopic and stochastic decision-making have important effects.

All three forms of governance significantly impacted forest cover dynamics, but they did so in very different ways. For example, a yearly incentive to conserve forest reduces the discrepancy between economic value of forested and deforested land. When the incentive is large this motivates landowners to conserve forest. However, behavioural responses mitigate the benefits of the incentive: as forest cover grows, expected utilities for deforestation are increasingly based on the decisions of a shrinking number of landowners who deforest, and if a single landowner decides to cut trees due to poor decision-making, other landowners may follow believing that the current value of deforested land is high. We note that this process is analogous to the dynamics of information cascades [36]. This can result in synchronized deforestation [23]. Hence, any mechanism that pushes forest cover to higher levels, such as an annual incentive to retain forest, can accelerate this effect. Thus, in addition to short-term memory and stochastic decision-making as a cause of synchronized deforestation [23], we find that governance as an annual incentive to retain forest can also induce synchronized deforestation, when decision-making is short-term, forest recovery is sufficiently rapid, and/or the incentive is sufficiently high. We note that the oscillations are sometimes on very long time scales and hence might not be observed in real populations without the obscuring effects of other influences such as changes in policy, demographics, or society.

The second form of governance we explored – a one-time penalty for deforestation – was generally found to be less subject to this instability. Large values of *g'* under this scenario counteract the effects of short-term memory (*α*), resulting in more stable dynamics; since the penalty is applied at the time of deforestation, the penalty never escapes the decision-making processes even when memory is short-term, meaning the current perceived value of deforested land is less than loss of ecosystem services of forested land. Landowners who are considering deforesting are forced to take the penalty into account, even when memory is short-term. Harper *et al.* [37] provide empirical data on Madagascan forest cover from 1950-2000 showing relatively stable trends in deforestation even after an emphasis was placed on environmental conservation (more penalties) in the late 1980s and early 1990s [27], [38]. The average rates of deforestation were 0.3% per year from the 1950s to the 1970s, which increased to 1.7% per year from the 1970s to 1990 and 0.9% per year from 1990 to 2000 [37].

The third form of governance – one-time incentive to reforest – was found to be least stable, since there was less incentive to maintain a patch in a forested state once the reforestation incentive had been received. In some cases, increased incentives actually decreased average forest cover and also caused oscillations.

Under yearly incentives for forest conservation and a one-time penalty for deforestation, as *g'* increases so does the average fraction of forested land. Despite its effects on stability when memory is short term, yearly incentives to maintain forest provide the greatest forest cover on average. Hence, when landowners adopt a sufficiently long-term perspective, governance as a yearly incentive for conservation may present the most effective form of governance. However, when landowners make myopic decisions, adding incentives increases the threat of synchronized deforestation. Even when average forest cover is higher, the extremely low values of forest cover reached during transient periods of deforestation may bring about other effects we have not addressed explicitly, such as soil erosion and species extinctions due to lack of habitat reliability.

The predictions of our model are reflected in the experiences with incentivization programs that have been established in various regions in recent decades [13], [39], [40]. For example, there is potential for corruption in any form of government, especially when economic incentives for reforestation are concerned [13]. The more corrupt a region is, the less likely incentives for reforestation or conservation are able to achieve their goal of maintaining stable forested landscapes [40]. Corruption pertains to the duration of memory, *α*, where shorter-term memory (higher *α*), would apply in jurisdictions where corruption is more rampant. When *b'* and *c'* are high, only the utility of the land matters for decisions, but when *b'* and *c'* are low, other considerations besides utility can influence decisions, and one such consideration is the effect of corruption in biasing the decision to deforest or reforest, even against what utility suggests is the best decision. Hence, the condition of high *α* (myopic decision-making), with the accompanying negative impacts on average forest cover and stability, describe more corrupt jurisdictions and lower quality governments more generally [15]. Introducing financial incentives in regions with corrupt governments or where little emphasis has been on forest conservation in years past does little to compensate for governance failures [14].

The effect of lower values of *b'* and *c'* is also reflected in other considerations that can enter decision-making which are not directly relevant to utility. For example, landowner perception of the political function of incentives may influence whether or not they accept them, or the incentives themselves may be used for political purposes. This has occurred in the Amazonas states, where some landowners view the payments for environmental services as a right or even as a sort of political bribery [41]. Corruption needs to be reduced, laws and regulations must be upheld and the government itself must show interest in forest management for environmental reasons and not just for received payment [14], [41]. The political situation in Brazil exemplifies the strong influence of government policy on deforestation rates [42], [43]. Forestry is an economic driver in Brazil thus forest conservation is not always a priority, especially when combined with unsecure land ownership [42], illegal logging [42], agricultural activities [44] and cattle ranching [45], [46]; as a result deforestation rates in the region have followed a boom bust cycle [42], [43]. From 1988–2007 the deforestation rate fluctuated between 11 000 km^2^ and 29 000 km^2^. Tax credits and incentives to a given sector determined deforestation activity [47].

In other respects our model does not capture effects that are relevant to incentive design in real populations. For example, although a penalty for deforestation yields the most stable forest cover in our model, penalties are rarely chosen as a primary method for deforestation mitigation in practice. This is partly because penalties can decrease social welfare, further impoverishing individuals and communities, such as in Madagascar and Taiwan [27], [28]. Burning forest is a traditional agricultural activity in Madagascar [27] and the legislation put forth to criminalize burning practices has resulted in increased deforestation caused by illegal burning in the form of protest [48]. The rise in deforestation rates mentioned above (ref. 37) was likely due to increased poverty, retaliation to the neo-colonial First Republic and a change in legislation that relaxed all regulations pertaining to natural resource use [38]. Our model is not socially structured and hence cannot address issues of social equity. Other authors have similarly identified the potential for counter-productive outcomes for REDD+ governance strategies involving heavy government intervention, suggesting that it may inadvertently cause effects such as “land grabs” of aboriginal territory [39]. Although Griffiths [39] is concerned with counter-productive feedbacks in social equity instead of forest cover stability, our model highlights how governance efforts can be undermined by behavioural feedback under certain conditions. In real populations farming is a significant contributor of forest degradation and a government incentive is not enough not to arrest deforestation for large-scale agricultural development; however incentives can curb the use of forested land developed for small and medium crops [13]. Our model does not consider the gains from cash crops since large agricultural development would be unaltered by incentives and small crops would not contribute significant gains to utility. Finally, the mechanism for synchronized deforestation may not be robust to adding other elements of decision-making, since the mechanism depends on the actions of a few individuals near a threshold.

In this model we do not distinguish between old growth forests and plantations. Without distinguishing between timber harvest from plantations and the clearing of natural forests, the impacts of PES on deforestation rates in Costa Rica are thought to be undervalued [11]. Neither Pfaff *et al*. [49] nor Sánchez-Azofeifa *et al*. [34] distinguish between harvesting timber from plantations and natural forest clearing, as a result their findings contradict those of ref. 35; Pfaff *et al*. found less than 0.25% of land enrolled in the PES program had reduced deforestation rates [49]. These studies question the effectiveness of forest regeneration programs (similar to our third scenario – one-time incentive for reforestation) and stress the implications of converting primary forests to plantations.

This work shows that the design of incentive programs intended to increase forest cover must carefully consider the possibility of behavioural feedbacks that can undermine well-intentioned policy in the long-term. Penalizing deforestation is predicted to be most successful at promoting stable forest cover, although issues of equity and social welfare need to be considered in any structure that penalizes deforestation. Hence, future work could explore strategies for optimizing the stability/equity trade-off, such as by combining deforestation penalties for wealthy landowners with conservation incentives for poor landowners. Coupled human-environment system models provide a useful framework for posing questions and thinking about the long-term effects of reforestation initiatives, especially in light of the multiple ecological and sociological factors that can influence their success. These models should be further developed through closer integration with empirical data and by tailoring them more closely to specific policy situations.

## Supporting Information

Supporting Information S1 **Classification of equilibrium and supporting stability matrices.** Description of the stable and unstable systems in our model using Jacobian Matrices (S1, S2) under yearly incentive for forest conservation and penalty for deforestation.
(DOC)
