# Supplementary material for: Carrot or Stick? Modelling How Landowner Behavioural Responses Can Cause Incentive-Based Forest Governance to Backfire
Source: PLoS One. 2013 Oct 30;8(10):e77735. doi: 10.1371/journal.pone.0077735 (PMC3813756; doi:10.1371/journal.pone.0077735)
Supplement: Supporting Information S1 — Classification of equilibrium and supporting stability matrices. Description of the stable and unstable systems in our model using Jacobian Matrices (S1, S2) under yearly incentive for forest conservation and penalty for deforestation. (DOC) [file pone.0077735.s001.doc]

**Supporting Information S1**

A stable equilibrium is one which the absolute value of all eigenvalues obtained from the Jacobian Matrix are less than 1. A stable system is classified into two categories: stationary-forested landscapes and stationary-deforested landscapes [23]. Stationary-forested landscapes occur if the density of forested land (*x*(*t*)) is greater than the density of deforested land (*y*(*t*)). Stationary-deforested landscapes occur if the density of deforested land (*y*(*t*)) is greater than the density of forested land (*x*(*t*)).

As noted by Satake *et al.*, an unstable equilibrium is classified into two categories: “the fluctuation near the equilibrium” and "synchronized deforestation” [23]. The stability of eigenvalues for conservation governance with yearly incentive and penalty for deforestation governance are found numerically using Matrices (S1, S2), since there is no analytic solution. Incentive for reforestation governance does not have a positive equilibrium value and the stability cannot be determined using the Jacobian Matrix. When the dominant eigenvalue is a negative real eigenvalue with a magnitude greater than 1, this form of instability is termed “the fluctuation near the equilibrium” as seen in Figures 3B and 5B, where *α* is small. For dominant eigenvalues that are complex conjugates with a magnitude greater than 1, we refer to this as “synchronized deforestation”. As seen in Figures 3C and 5C for large values of *α* create oscillations with large amplitudes, (otherwise known as synchronized deforestation).

*Yearly incentive for forest conservation:*

(S1)

*Penalty for deforestation:*

(S2)
